# Effect of Kegel Pelvic Floor Muscle Exercise Combined with Clean Intermittent Self-catheterization on urinary retention after radical hysterectomy for cervical cancer

**DOI:** 10.12669/pjms.38.3.4495

**Published:** 2022

**Authors:** Jingjing Zong, Minghui You, Chen Li

**Affiliations:** 1 Jingjing Zong, Department of Reproductive Medicine, Baoding First Central Hospital, Baoding, Hebei, 071000, China; 2 Minghui You, Department of Anoenterology, Shandong Second Provncial Generat Hospital (Shandong Provincial ENT Hospital), Jinan, Shandong, 250000, China; 3 Chen Li, Department of Gynecology and Obstetrics, Baoding First Central Hospital, Baoding, Hebei, 071000, China

**Keywords:** Clean intermittent self-catheterization, Cervical cancer, Kegel pelvic floor muscle exercise, Radical hysterectomy, Urinary retention

## Abstract

**Objectives:**

To investigate the effect of Kegel pelvic floor muscle training combined with clean intermittent self-catheterization on patients with cervical cancer, and to analyze the risk factors affecting urinary retention.

**Methods:**

A total of 166 patients with cervical cancer admitted to our hospital from October 2016 to December 2019, all of whom received radical resection of cervical cancer, were divided into two groups according to the random number table method: the observation group and the control group, with 83 cases in each group. The control group underwent clean intermittent self-catheterization, while the observation group underwent Kegel pelvic floor muscle exercise combined with clean intermittent self-catheterization. The catheter replacement rate, bladder residual urine volume, self-perceived burden scale (SPB), Kolcaba general comfort questionnaire (GCQ), incidence of urinary tract infection, and urinary retention after catheter removal were compared between the two groups. Logistics regression analysis was utilized to analyze the risk factors affecting urinary retention.

**Results:**

The incidence of catheter replacement, urinary retention, dysuria and bladder residual urine volume in the observation group were significantly lower than those in the control group (P<0.05). Postoperative SPB score of the two groups decreased significantly, while the GCQ score increased significantly. Postoperative SPB score of the observation group was significantly lower than that of the control group, while the GCQ score was significantly higher than that of the control group (P<0.05). Statistically significant differences can be observed in the comparison of catheter indwelling time, urinary tract infection, surgical incision infection and surgical margin between the two groups (P<0.05). Logistic regression analysis showed that catheter indwelling time, urinary tract infection, surgical incision infection and surgical margin were independent risk factors affecting urinary retention (P<0.05).

**Conclusions:**

Catheter indwelling time, urinary tract infection, surgical incision infection and surgical margin are the risk factors for postoperative urinary retention in patients with cervical cancer. With Kegel pelvic floor muscle exercise combined with clean intermittent self-catheterization, a variety of benefits can be realized, such as improved bladder function, reduced incidence of urinary tract infections and urinary retention, as well as increased patient comfort.

## INTRODUCTION

Cervical cancer, as a common clinical gynecological malignant tumor, is second only to breast cancer in the incidence of gynecological tumors. Early cervical cancer presents no typical symptoms and is easily overlooked, leading to invasion and metastasis of cervical cancer.^1^,^2^ Currently, radical resection of cervical cancer is used as the principal mean for the treatment of early cervical cancer (stage IA to IIA). During the surgical resection of cervical cancer, the pelvic autonomic nerve will be destroyed and part of the nerves that innervate the bladder will be cut off, leading to bladder contraction and sensory dysfunction, as well as urination disorders and urinary retention[3]. After the urinary catheter is removed, the patient is unable to urinate successfully, which may lead to urinary tract infection and renal insufficiency in severe cases. In the process of clinical nursing, patients are usually instructed by nurses to carry out intermittent clamping training about three day prior to catheter removal to ensure smooth urination after catheter removal. However, practice shows that intermittent pinching training alone is not effective, with unsatisfactory bladder recovery effects for patients. For this reason, new nursing methods are urgently needed to be developed in the clinic to solve the problems of bladder function recovery and urinary retention after radical resection of cervical cancer.^3^,^4^

With clean intermittent self-catheterization, patients can remain relatively free of catheterization and maintain a moderate intravesical pressure, which is conducive to reducing the risk of complications and the recovery of bladder function. Therefore, this surgical method is widely applied in clinical practice. Pelvic floor muscles surround the urethra, vagina, etc., support pelvic and abdominal organs, and have a close bearing on the urination function. It has been observed in some studies that pelvic floor muscle exercise boasts various benefits such as improving pelvic floor muscle contraction and diastolic tension, enhancing urinary continence capability, and accelerating the recovery of bladder function.^5^,^6^

There are few reports on the combined effect of pelvic floor muscle exercise and clean intermittent self-catheterization in patients with cervical cancer after surgery. The purpose of this study was to investigate the effects of Kegel pelvic floor exercise combined with self-cleaning intermittent catheterization on postoperative patients with cervical cancer, and to elucidate the risk factors for urinary retention.

## METHODS

A total of 166 patients with cervical cancer admitted to our hospital from October 2016 to December 2019, all of whom received radical resection of cervical cancer, were divided into two groups according to the random number table method: the observation group and the control group, with 83 cases in each group, aged 30±56 years old. The sample size required for each group is calculated by the formula.


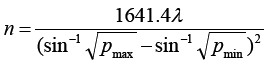


All patients signed an informed consent form, and this study was conducted under the approval of the Ethics Committee of our hospital.(Ref. approved on March 31, 2021)

### Inclusion criteria:

- Patients who have been diagnosed as cervical cancer by pathology and imaging tests and whose clinical stage is Ia-IIa;^7^
- Newly treated patients who have not received radiotherapy, chemotherapy, or surgery;
- Patients with a hospital stay of no less than 48h and postoperative catheter indwelling of no less than three days;
- Patients with reading, writing and cognitive capabilities and able to independently complete the postoperative questionnaire.

### Eexclusion criteria:

- Patients with preoperative urinary system infections, bladder tumors, urinary calculus and other urinary system diseases;
- Patients with dysfunction of the heart, kidney, liver and other important organs;
- Patients who received radiotherapy or chemotherapy after surgery.

### Nursing methods

Patients in the control group received clean intermittent self-catheterization. The specific process is as follows: Patients cleaned their hands and urethral orifice under running water according to standard procedures, then picked up the rear end of the catheter and moistened it with warm boiling water near the tip of the catheter. Patients then slowly inserted the catheter into the urethra with thumb and forefinger until the urine flowed out and continued to insert 1-2cm. Gently pressed the bladder after the outflow stops, and then slowly pulled out of the catheter after confirming that there is no urine outflow. After the procedure, the catheter was rinsed with cold water, dried in the shade and stored in a neat storage box. The time interval of urethral catheterization was set according to patients’ residual urine volume, usually 4-6hour and 4-6 times a day. Patients were instructed to lie on their sides, turn over, and get out of bed regularly, and received education on urinary incontinence, urinary retention and other related complications, such as the replacement of drainage bag, the maintenance of catheter patency, drinking instruction, clamping and catheter removal time. At the same time, responsible nurses were also required to provide acute psychological care and guidance to the patients, and pay close attention to the abnormal state (depression, anxiety, etc.) of the patients.

Patients in the observation group underwent Kegel pelvic floor exercise on the basis of self-cleaning intermittent catheterization, that is, patients were instructed to perform contraction training on the abdominal muscles, vulva and pelvic floor muscles. Patients were instructed on the procedure of Kegel pelvic floor muscle exercise 3d before surgery, and diastolic and contractile exercises of the vagina, urethra and anal sphincter were performed on the 4d after surgery while lying in bed. Patient were supine with legs flexed apart. When inhaling, the perineum and anus were contracted forcibly, lasting about 10 seconds, and when exhaling, it was relaxed about 10s. The above actions were repeated for about 20 minutes, with an interval of 5-10s between each time, three times a day. During the hospitalization, the responsible nurse provided regular guidance and training. After discharge, regular telephone follow-up was conducted to 14 days after surgery.

### Self-perceived burden

The self-perceived burden (SPB) scale^8^ was adopted for evaluation. The higher the score, the more serious the self-perceived burden.

### Kolcaba general comfort questionnaire (GCQ)^9^ was adopted, which was divided into four dimensions

physiology, spirit, psychology, social culture and environment. A 4-point Likert scale was used for scoring, which was divided into 4 grades from 1 to 4 points, of which four points indicate strongly agree and 1 point indicates strongly disagree. The higher the score, the more comfortable it would be.

Four hours after the catheter was removed, the patient was instructed to empty the bladder, and the residual urine volume of the patient’s bladder was measured by three-dimensional color ultrasound. And the occurrence of complications such as urinary retention, urinary incontinence, urinary catheter reset, and urinary tract infection were recorded. In this study, urinary retention was defined as bladder residual urine volume greater than 100ml after forced urination 4 hours after catheter removal. In case of more than 100mL residual urine on the day of extubation, the catheter needs to be reset, otherwise, the extubation is successful.

### Statistical Analysis

All the data of this study were statistically analyzed by SPSS 20.0 software, and the measurement data were expressed in the form of number of cases (percentage). Statistical analysis was performed using the *χ^2^* test, and measurement data were expressed as mean ± standard deviation (*x¯*). Statistical analysis was performed using the *t* test, and data at different time points were compared using repeated measurement analysis of variance. In univariate analysis, the statistically significant factors were included in the multivariate logistic regression analysis. P<0.05 indicates a statistically significant difference.

## RESULTS

No significant difference can be seen in the comparison of baseline information such as age, duration of surgery and length of hospital stay between the two groups (P<0.05), Table-I.The incidence of catheter replacement, urinary retention, dysuria and bladder residual urine volume in the observation group were significantly lower than those in the control group, with statistically significant differences (P<0.05). Table-II.

**Table-I:** Comparison of baseline information (n, *x*¯±s).

Group	n	Age (Years Old)	Duration of Surgery (min)	Length of Hospital Stay (d)	Cancer Type		Clinical Stage		

					Squamous cell carcinoma	Adenocarcinoma	Stage IA	Stage IB	Stage IIA
Observation Group	83	42.44±3.53	143.44±23.53	12.94±2.13	58	25	17	39	27
Control Group	83	42.75±3.91	140.75±21.91	13.45±2.01	55	28	20	37	26
t/χ^2^		0.536	0.762	1.587	0.249			0.315	
P		0.593	0.447	0.115	0.618			0.854	

**Table-II:** Comparison of the occurrence of complications after removing the catheter between the two groups (n, *x*¯±s).

Group	n	Catheter replacement (case)	Urinary retention (case)	Urinary incontinence (case)	Dysuria (case)	Bladder residual urine volume (mL)
Observation Group	83	14	13	4	9	46.79±10.20
Control Group	83	25	26	7	19	67.37±18.47
*t/χ^2^*	-	4.055	5.664	0.876	4.296	8.886
*P*	-	0.044	0.017	0.349	0.038	<0.001

Postoperative SPB scores of the two groups decreased significantly, while the GCQ scores increased significantly. Postoperative SPB score of the observation group was significantly lower than that of the control group, while the GCQ score was significantly higher than that of the control group, with statistically significant differences (P<0.05). Table-III.

**Table-III:** Comparison of preoperative and postoperative SPB scores and GCQ scores (n, *x*¯±s).

Group	n	SPB scores			GCQ scores		

		3d before surgery	3d after surgery	10d after surgery	3d before surgery	3d after surgery	10d after surgery
Observation group	83	49.38±2.91	43.67±3.27^ab^	37.59±2.78^ab^	46.56±3.21	56.48±3.42^ab^	74.58±4.26^ab^
Control group	83	48.87±2.82	45.76±3.16^a^	40.54±3.02^a^	47.17±3.42	52.48±3.38^a^	68.69±4.17^a^
Inter-group		F1=16.58;P2=0.000			F1=25.469;P1=0.000		
Different time		F2=11.362;P1=0.000			F2=18.453;P1=0.000		
Inter-group × Different time		F3=37.753;P3=0.000			F3=48.713;P2=0.000		

***Note:*** A means P<0.05 compared with 3d before surgery, and b means P<0.05 compared with the control group.

Thirty-nine patients developed urinary retention after surgery and were divided into two groups: the urinary retention group and the normal group. Statistically significant differences can be observed in the comparison of catheter indwelling time, urinary tract infection, surgical incision infection and surgical margin between the two groups (P<0.05). However, no significant difference was observed in the comparison of general data such as age, duration of surgery and intraoperative blood loss. Table-IV.

**Table-IV:** Comparison of baseline information.

Group	Urinary retention group (39 cases)	Normal group (127 cases)	t/χ^2^	P
Age (years old)	43.23±3.79	42.40±3.65	1.232	0.220
Duration of surgery (min)	145.23±22.28	141.13±21.85	1.020	0.309
Intraoperative blood loss (mL)	85.32±20.23	81.53±17.45	1.142	0.225
Length of hospital stay (d)	13.71±2.18	13.04±2.05	1.767	0.079
Cancer types			0.370	0.543
Squamous cell carcinoma	25	88		
Adenocarcinoma	14	39		
Clinical stage			0.789	0.674
Stage Ia	7	30		
Stage Ib	20	56		
Stage IIa	12	41		
Catheter indwelling time (d)	11.71±2.38	10.04±2.12	4.179	<0.001
Urinary tract infection	12	20	4.326	0.038
Surgical incision infection	18	26	10.102	0.002
Surgical margin			4.511	0.034
Subextensive hysterectomy	23	97		
Extensive hysterectomy	16	30		

Logistic regression analysis, taking urinary retention as the dependent variable (1=Yes, 0=No), and catheter indwelling time, urinary tract infection, surgical incision infection and surgical margin as the independent variables, showed that catheter indwelling time, urinary tract infection, surgical incision infection and surgical margin were independent risk factors affecting urinary retention (P<0.05).Table-V.

**Table-V:** Logistic regression analysis affecting urinary retention.

Affecting factors	β (regression coefficient)	SE	Wald X²	P value	OR	95%CI	
Catheter indwelling time	0.526	0.147	12.804	0.000	1.692	1.269	2.257
Urinary tract infection	0.726	0.322	5.083	0.024	2.067	1.100	3.885
Surgical incision infection	1.131	0.367	9.497	0.002	3.099	1.509	6.362
Surgical margin0	0.646	0.287	5.066	0.024	1.908	1.087	3.349

## DISCUSSION

Radical resection of cervical cancer is prone to various complications due to large anatomical variation in the surgical site and large surgical wound area, among which urinary retention is a common complication. For this reason, improving intraoperative skills, reducing nerve damage and strengthening postoperative nursing care are of great importance for reducing the risk of complications.^10^-^12^ Clean intermittent catheterization, as a scientific method of urinary tract management, has been widely applied in clinical practice. When performing clean intermittent catheterization, a urinary catheter is placed from the urethra into the bladder regularly in a sterile environment, so as to empty the urine regularly.^13^,^14^ With clean intermittent self-catheterization, the self-care ability of patients can be improved, and the psychological and physical stress caused by the indwelling catheter can be avoided, which is conducive to the recovery of bladder function and the reduction of the risk of complications (such as infection and urinary incontinence).

When performing clean intermittent self-catheterization, patients are required to strictly control the clean environment before catheterization, clean their hands, wash the perineum, and strictly implement a drinking plan to ensure that the bladder is regularly filled, emptied, and discharged regularly, which is conducive to reducing or preventing urinary tract infections.^15^,^16^ Nevertheless, prolonged postoperative indwelling of the urethral catheter may affect the function of the urethral sphincter, weaken bladder tension and detrusor contraction, increase the pressure of the bladder and urethra during urination, and affect normal urination, thereby increasing the incidence of complications such as urinary retention and dysuria.^17^ It is shown in this study that the incidence of catheter replacement, urinary retention, and dysuria in the observation group was significantly lower than that in the control group, indicating that Kegel pelvic floor muscle exercise based on clean intermittent self-catheterization can effectively reduce the risk of complications. Which is consistent with the results of previous studies. In the event of catheter replacement and prolonged indwelling of catheter, a series of adverse effects will be caused to patients, such as affecting patients’ confidence and feeling of voluntary urination after indwelling the catheter, as well as delaying the recovery of bladder function. During Kegel pelvic floor muscle exercises, a variety of benefits can be brought about via repeated and autonomous relaxation and contraction of pelvic floor muscles, such as promoted pelvic floor blood circulation, enhanced pelvic floor muscle tension and contractile and diastolic ability of levator ani muscle and distal urethral sphincter, improved urinary continence capability to avoid the risk of urinary incontinence, accelerated recovery of bladder function.^18^ The results of this study also show that, bladder residual urine volume in the control group was significantly higher than that in the observation group. Postoperative SPB score of the observation group was significantly lower than that of the control group, while the GCQ score was significantly higher than that of the control group, indicating that Kegel pelvic floor muscle exercise combined with clean intermittent self-catheterization can reduce the burden on patients from mental and psychological aspects, prevent urinary system diseases complicated by cervical cancer surgery, and solve bladder dysfunction, so as to avoid infection and promote bladder function recovery, protect kidney function, and improve the quality of life of patients and nursing satisfaction. Previous studies have also found that kegel exercise can significantly improve bladder function, pelvic floor strength and quality of life in patients with urinary incontinence.^19^

During the radical operation of cervical cancer, important organs such as ureter, pelvic cavity and bladder are involved, which may easily damage related organs and connecting nerves, resulting in urinary retention and seriously affecting the quality of life of patients. Logistic regression analysis in this study show that catheter indwelling time, urinary tract infection, surgical incision infection, and surgical margin are independent risk factors affecting postoperative urinary retention in patients with cervical cancer (P<0.05). The extent of surgical resection has a bearing on neurogenic bladder dysfunction. Extensive hysterectomy requires complete removal of the ligaments around the cervix, but with a large surgical margin and a damaged pelvic autonomic nerve. As a result, the innervation function of the bladder is affected, the properties of the elastic muscle fibers of the bladder wall are reduced, the sphincter is relaxed, and the bladder is paralyzed, etc.^20^, thus increasing the risk of urinary retention. Prolonged catheter indwelling can not only affect bladder tension and urinary continence ability, but also increase the risk of urinary tract infection and aggravate dysuria, thus increasing the risk of urinary retention. Surgical incision infection can lead to urinary tract infection, which in turn causes the inflammatory reaction of the detrusor muscle, leading to edema, weakening the tension of detractor muscle, and thus easily causing urinaryretention.^21^ Urinary retention is caused by a variety of factors. Therefore, targeted nursing interventions are needed to reduce the risk of occurrence. First of all, Kegel pelvic floor muscle exercises, abdominal muscle exercises, etc. can be carried out to enhance the ability of pelvic floor muscle groups to contract and urinary continence. Secondly, clean intermittent catheterization care should be performed after surgery to strengthen the cleaning and care of the perineum and urethral orifice. Soft urethral catheters should be selected to avoid urethral mucosal injury, smooth drainage should be maintained, and urinary bags should be replaced regularly. In addition, patients should drink more water to increase urinary excretion. All of these interventions can reduce the risk of urethral infections. Based on the pathophysiological state of the patients, comprehensive nursing can reduce the risk of postoperative urinary retention by selecting the appropriate timing of catheterization and extubation, improving the operational skills of physicians, reducing the resection margin, preserving neurosurgical resection and so on.

### Limitations of this study

The number of subjects included in this study is limited, so the conclusions drawn may not be very convincing. In addition, there was no significant statistical difference in the incidence of urinary incontinence after extubation between the two groups, which may be due to the small number of study cases. More time and effort will be needed to refine the data in the future in the hope of reaching a stronger conclusion.

## CONCLUSION

Catheter indwelling time, urinary tract infection, surgical incision infection and surgical margin are the risk factors for postoperative urinary retention in patients with cervical cancer. With Kegel pelvic floor muscle exercise combined with clean intermittent self-catheterization, a variety of benefits can be realized, such as improved bladder function, reduced incidence of urinary tract infections and urinary retention, as well as increased patient comfort.

### Authors’ Contributions:

**JZ &** **IY:** Designed this study and prepared this manuscript, and are responsible and accountable for the accuracy or integrity of the work.

**JZ &** **MY:** Collected and analyzed clinical data.

**CL:** Significantly revised this manuscript.
